# ASO Author Reflections: Consideration of a Modified Classification System for Gastric Cancer Based on Lauren Subtypes

**DOI:** 10.1245/s10434-018-6976-6

**Published:** 2018-10-29

**Authors:** Lin-Yong Zhao, Jian-Kun Hu

**Affiliations:** Department of Gastrointestinal Surgery and Laboratory of Gastric Cancer, State Key Laboratory of Biotherapy, West China Hospital, Sichuan University and Collaborative Innovation Center for Biotherapy, Chengdu, Sichuan Province China

## Past

Gastric cancer (GC), as one of the most common cancers and leading causes of cancer-related mortality globally, is a heterogeneous malignance, and the analysis of its molecular and clinical characteristics has been complicated by histological and etiological heterogeneity. Although the Lauren classification (LC) has been widely used as pivotal histologic subtypes to understand the GCs since it was proposed in 1965, Shah implemented with a modified Lauren classification (mLC) system on the basis of anatomic location and epidemiology, as well as histopathologic classification, and found that each subtype could be distinguished from each other by differentially expressed genes.^1^ However, no unanimous consensus has been reached so far on whether the mLC system is superior to LC system. This study aimed to clarify the clinical significance of the mLC and to compare it with the LC for GC patients from three institutions.^2^

## Present

The modified Lauren classification (mLC) in our study was found to be closely related to the depth of tumor invasion (T), lymph node metastasis (N), and distant metastasis (M), implying that mLC was likely to be associated with biological behaviors and aggressive features.^2^ Besides, the mLC rather than the LC system was illustrated to be an independent prognostic factor, with a better prognostic discriminatory ability and predictive accuracy than LC system. Therefore, compared with the LC system, the mLC could be considered a more reliable prognostic factor and may improve the prognostic discriminatory ability and predictive accuracy for gastric cancer patients. We hope that there will be more specific findings and evidence provided in larger sample-sized studies about the mLC system, which evaluated its clinical utility and made a comparative analysis with the LC system.

## Future

Subtyping gastric cancer (GC) is not only to discriminate its survival prediction but to observe its heterogeneity in response to treatments, because that one-size-fits-all approach to GC therapy has no longer been utilized. Personized comprehensive multidiscipline therapy should be advocated widely. Apart from the histopathologic or anatomical classifications, such as WHO subtypes, Lauren classification, Borrmann subtypes, etc., molecular screening and optimized therapy according to the GC molecular classifications have to be taken into account, thus making the development of TCGA and ACRG subtypes for GCs, from the genomic and transcriptional level, respectively.^3^^,^^4^ All of these confirmed that GC is a heterogeneous disease both in molecular biology and histopathologic morphology. As a consequence, an integrated classification system will be preferred and utilized in the future. Therefore, further studies are needed to create a consensus with respect to different ways of classifications and their clinical relevance for widespread use.
